# Boolean modeling reveals that cyclic attractors in macrophage polarization serve as reservoirs of states to balance external perturbations from the tumor microenvironment

**DOI:** 10.3389/fimmu.2022.1012730

**Published:** 2022-12-05

**Authors:** Ugo Avila-Ponce de León, Aarón Vázquez-Jiménez, Meztli Matadamas-Guzmán, Osbaldo Resendis-Antonio

**Affiliations:** ^1^ Programa de Doctorado en Ciencias Biológicas, Universidad Nacional Autónoma de México, Ciudad de México, Mexico; ^2^ Human Systems Biology Laboratory, Instituto Nacional de Medicina Genómica (INMEGEN), Ciudad de México, Mexico; ^3^ Coordinación de la Investigación Científica – Red de Apoyo a la Investigación - Centro de Ciencias de la Complejidad, Universidad Nacional Autónoma de México, Ciudad de México, Mexico

**Keywords:** macrophage, cycle attractors, Boolean models, systems immunology, gene regulatory network, cancer immunology

## Abstract

Cyclic attractors generated from Boolean models may explain the adaptability of a cell in response to a dynamical complex tumor microenvironment. In contrast to this idea, we postulate that cyclic attractors in certain cases could be a systemic mechanism to face the perturbations coming from the environment. To justify our conjecture, we present a dynamic analysis of a highly curated transcriptional regulatory network of macrophages constrained into a cancer microenvironment. We observed that when M1-associated transcription factors (STAT1 or NF-κB) are perturbed and the microenvironment balances to a hyper-inflammation condition, cycle attractors activate genes whose signals counteract this effect implicated in tissue damage. The same behavior happens when the M2-associated transcription factors are disturbed (STAT3 or STAT6); cycle attractors will prevent a hyper-regulation scenario implicated in providing a suitable environment for tumor growth. Therefore, here we propose that cyclic macrophage phenotypes can serve as a reservoir for balancing the phenotypes when a specific phenotype-based transcription factor is perturbed in the regulatory network of macrophages. We consider that cyclic attractors should not be simply ignored, but it is necessary to carefully evaluate their biological importance. In this work, we suggest one conjecture: the cyclic attractors can serve as a reservoir to balance the inflammatory/regulatory response of the network under external perturbations.

## Introduction

Understanding how transcription factors (TFs) regulate and orchestrate gene expression in a cell has been an area of interest since the early stages of genomics research. In recent years, a variety of next-generation sequencing technologies have emerged, which have opened a new window to characterize the genetic regulatory mechanism, even at the single-cell level (1). Data generated from these technologies have been fundamental to discover the mechanical regulation of transcriptional factors creating databases and building high-quality gene regulatory networks at a genome scale (2–5). The gene regulatory interactions in an organism can be visually summarized in a network, whose topological and dynamical analysis elucidates their organization and feasible phenotypes (6–8). One approach to analyzing the dynamic portrayal of a regulatory network is the so-called Boolean model, which allows for an exploration of how the transcriptional regulatory network (TRN) responds to changes in the microenvironment. Boolean modeling assumes that the expression of genes, transcriptional factors, or signal components can take binary states: on (1) or off (0). The dynamical behavior of each node is determined through logical rules that describe the regulatory mechanisms over the gene (9). It is important to set the logic such that it does not depend on kinetics parameters or chemical concentration. Having selected an initial state and applied the Boolean rules synchronously or asynchronously for each node, the dynamic of the network is obtained until it reaches a steady-state behavior. Notably, despite its simplicity, this approach has proven to reproduce phenotype states in a variety of complex systems (8, 10). Although Boolean models cannot show the continuum behavior of variables over time and concentration having a dichotomous response, they give meaningful insights into the system steady states. In a TRN, they represent the time-invariant phenotype states. The steady states may be a single node (fixed-point attractors) or states that cycle among a set of specific nodes (cyclic attractors). The attractors are of great importance because they represent the long-term behavior of the Boolean model and are potentially associated with cellular phenotypes (11, 12). Most dynamical analyses of Boolean models explore the steady state of fixed-point attractors using synchronic simulation as a mathematical approach, most of the time neglecting their cyclic attractors. The period of cyclic attractors is one fundamental variable to distinguish and classify them, which is an integer indicating the number of states visited before recovering the initial state. Even though these analyses have significantly contributed to a variety of TRN, the question of whether the cyclic attractors have or do not have biological interpretation remains.

To evaluate the importance of cycle attractors, we will focus on an essential immune cell with a plethora of phenotypes based on what is present in the microenvironment, macrophages. These cells can balance between an anti-inflammatory and a pro-inflammatory response because they can transform from M1 to M2 phenotypes through intermediate phenotypes (13–15). Understanding the dynamics of polarization of the macrophages, especially those associated with oscillatory behavior, is crucial to understand the inflammatory response in cancer and COVID-19. Notably, some experimental studies stress the remarkable role that cyclic activation of key cytokines plays in regulating the cellular microenvironment through the anti/pro-inflammatory function of the macrophages (16). In addition, recent single-cell experiments and theoretical studies make evidence of the functional relevance of the heterogeneity and cyclic activation in macrophage polarization (17, 18). These and other facts suggest that the cyclic behavior of macrophage polarization seems to be an essential feature influencing biological function. In this perspective article, we theoretically studied the behavior of cyclic attractors and sketch their possible biological implications. To explore the properties of cyclic attractors, we used a high-quality gene regulatory network of macrophage polarization in a tumor microenvironment (TME) used in (8) and evaluated their dynamics under the synchronous updating scheme. In addition, we obtained the cell fate map for the synchronous updating scheme, which modifies the node of the attractor with the opposite value. For instance, if the value is 0, we perturbed it with 1 and evaluated the dynamics of the said perturbation. Our analysis supplies evidence that cycle attractors retrieved from Boolean dynamics behave as reservoirs to restore the population that was lost when the perturbation occurred when a steady-state attractor is subject to a perturbation in a TF associated with a specific phenotype. Despite this conjecture requiring experimental validation, we highlight that these cyclic states in macrophages can have a remarkable role to sustain cancer phenotype, a hypothesis that calls to not neglect their presence and rethink their role in a biological context.

## Current perspective of macrophage polarization Boolean models

### Boolean model of the GRN associated with the polarization of a macrophage

We focused our discussion on explaining the biological consequences of the cycle attractors because in most Boolean models they are ignored. To this end, we focus our attention to a recent GRN built to explore the relationship between macrophages and cancer cells (8). Previous studies have shown that the TME is a decisive factor to trigger the specific phenotype of the macrophage (19–22). At the network level, TFs regulate the expression of other TFs that eventually induce a change in the macrophage behavior based on the stimuli (23, 24). Depending on the stimuli, monocytes will differentiate into two types of macrophages: classically activated macrophages (M1) and alternatively activated macrophages (M2) (25). M1 macrophages are implicated in a pro-inflammatory response by secreting factors [such as IL-1*β*, IL-6, IL-12, IL-23, TNF–*α*, and some chemokine ligands like CCL2 and CCL3, among others (26, 27)] that will eliminate tumor cells. Meanwhile, M2 macrophages are associated with the proliferation and repair of tumor cells by secreting anti-inflammatory mediators like IL-10, TGF-*β*, and vascular endothelial growth factor (VEGF), among others (27, 28). M2 macrophages also have the ability to secrete chemokine ligands like CCL17 and CCL22, which have the ability to affect the immune infiltration in a TME (29, 30). A variety of papers have modeled macrophage polarization using a Boolean discrete approach (8, 31–33); however, most of them have ignored the dynamics of cycle attractors (8, 33). To our knowledge, the work from Ordaz-Arias and colleagues is the first paper that has evaluated the importance of cycle attractors for macrophage plasticity in response to the microenvironment (17). They concluded that cyclic attractors are necessary to regulate the production of cytokines by macrophages based on a response in a changing microenvironment. To enhance the importance of the cyclic attractors, here we suggest that cyclic attractors serve as a reservoir to respond with phenotypes that counteract hyper-inflammatory or hyper-regulated states. Interestingly, the M1/M2 paradigm is very similar to the Th1/Th2 mice response. M1 or Th1 involves the activations of phenotypes associated with cytotoxic capacities. Meanwhile, M2 or Th2 is implicated with phenotype towards a regulatory behavior (34, 35). Evaluating the M1/M2 axis as an integrated process reveals the relevance of the balance between cytotoxic and regulatory behavior. Thus, a distress in the equilibrium might bend the response, leading to a systemic malfunction like chronic inflammation.

### Cycle attractors are essential for explaining macrophage adaptation in a tumor microenvironment

**Figure 1:**
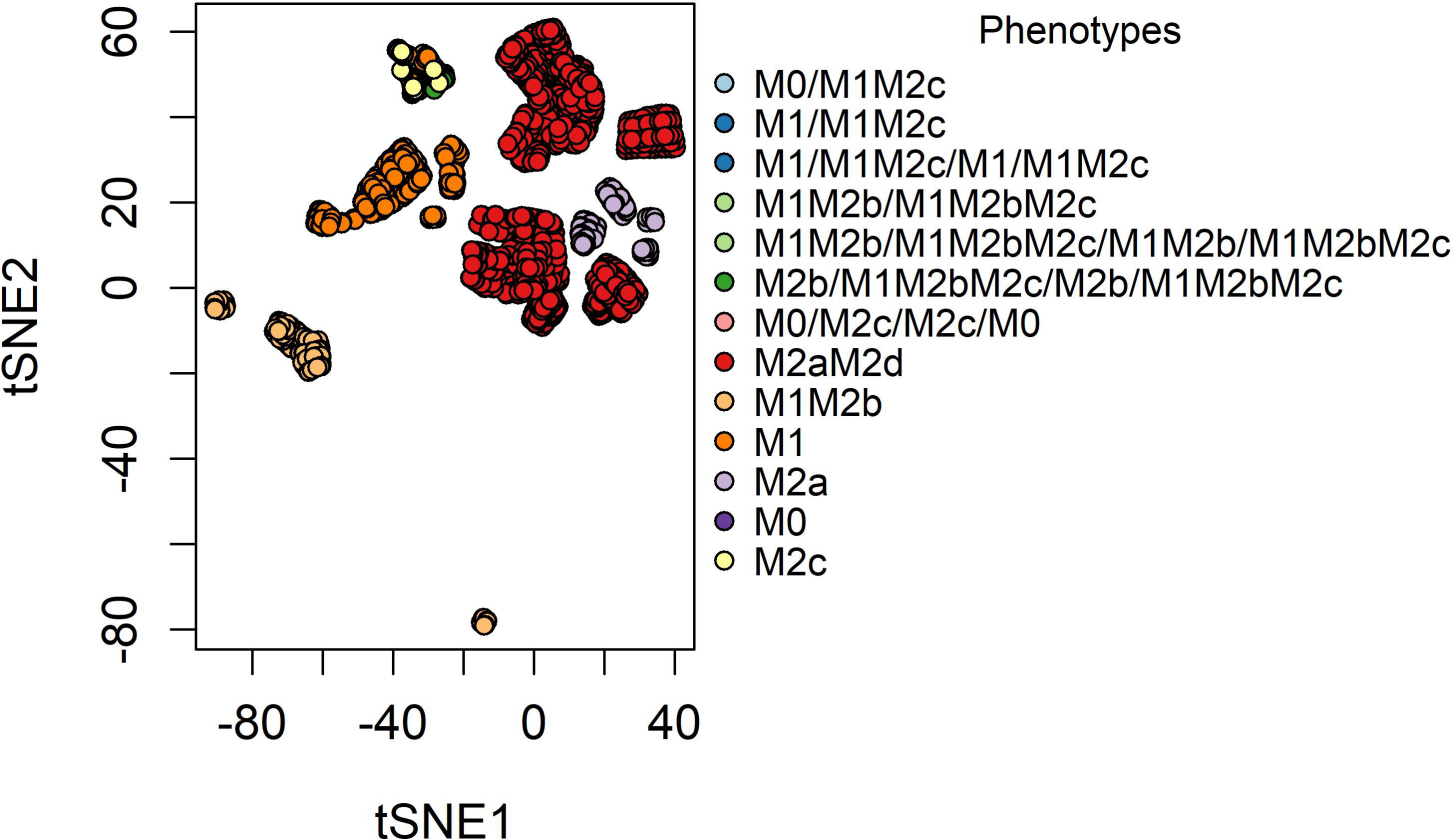
The t-SNE plot of the phenotypes was obtained from our network of macrophage polarization. The attractors were handed a point in an *x*-*y* plane based on the expression of 0’s and 1’s, and attractors with the same combination of 0’s and 1’s were placed nearby.

The reconstructed macrophage polarization network in a TME was meticulously simulated as a dynamical Boolean model using the synchronous updating scheme until the systems reach their equilibrium states (attractors) (**Figure 1**). To identify similarities among attractors, we have made a bi-dimensional map using the distributed stochastic neighbor embedding algorithm (36). As a result, we obtained 27 attractors, of which 19 were simple attractors and the remaining were cycle attractors. To associate the attractors with biological functionality, we labeled them based on six possible phenotypes previously described in reference (8). In this research, we defined a macrophage based on the possible functional behavior in a TME, i.e., tumor-eliminating or tumor-promoting macrophages (37). In our simulations, we obtained three types of attractors: pure fixed point, hybrid fixed point, and cycle attractors. Pure fixed-point attractors are the ones that only present one phenotype based on the experimental evidence of macrophage phenotypes (38, 39). Meanwhile, hybrid fixed-point attractors are phenotypes with at least one macrophage phenotype as in (8). Of the 19 pure fixed-point attractors, 10 are associated with tumor-promoting behavior and the rest are implicated with hybrid functions (labeled as hybrid fixed-point attractors) integrating tumor-eliminating and -promoting properties. From here, we conclude that, in a TME, the balance between pro- and anti-inflammatory processes is tilted toward the anti-inflammatory tumor-promoting properties (20, 40). On the other hand, all the cycle attractors have a hybrid phenotype having more than two macrophage phenotypes expressed simultaneously in each state. Only two cycle attractors have a period of two and the rest have a period of four. From a practical point of view, we can suggest the functionality of each attractor and the regulators that they involve in the network. The two-period cycle attractor M0/M1M2c is associated with the activation of AP-1, STAT3, and NF-*κ*B. Because the latter TFs are activated, the associated cytokines that may be secreted in the TME are IL-6, IL-1*β*, and IL-10. The four-period cycle attractor M1/M1M2c/M1/M1M2c is associated with the expression of a pleiotropic IL-6, which is implicated in acute inflammation and tumor proliferation. IL-6 can activate AP-1 and STAT3 TFs (16); both factors are implicated with dual behaviors in cancer. IL-6 is associated with a back-and-forth cycle between tumor elimination and tumor promotion. Experimental evidence has shown that it is capable of promoting positive feedback between breast cancer and tumor-associated macrophages for metastasis and proliferation (41, 42). The two- and four-degree cycle attractors M1M2b/M1M2bM2c and M1M2b/M1M2bM2c/M1M2b/M1M2bM2c are dictated by the expression of STAT1, AP-1, NF-*κ*B, and STAT3. M2b/M1M2bM2c/M2b/M1M2bM2c is maintained in a cycle due to the action of a pro-inflammatory IL-1*β*.Like IL-6, IL-1*β* has two sides to a story: tumor promotion and tumor inhibition. These cytokines are connected because they are associated with secreting cytokines and tumor-promoting factors to enhance breast cancer cells’ capacity to thrive and metastasize (43). However, they have a tumor-inhibiting capacity because they promote an M1 tumor cytotoxic behavior and recruit Th1 and Th17 as anti-tumorigenic effects (44). Another cycle attractor is M0/M2c/M0/M2c, which is associated with the expression of STAT3 caused by IL-10 or IL-6. Interestingly, when any of these cytokines are not present, the M2c phenotype goes back to a monocyte with M2c secretion capacities.

### Perturbing key molecular components is associated with transitions to cycle attractors that may act as reservoirs

**Figure 2:**
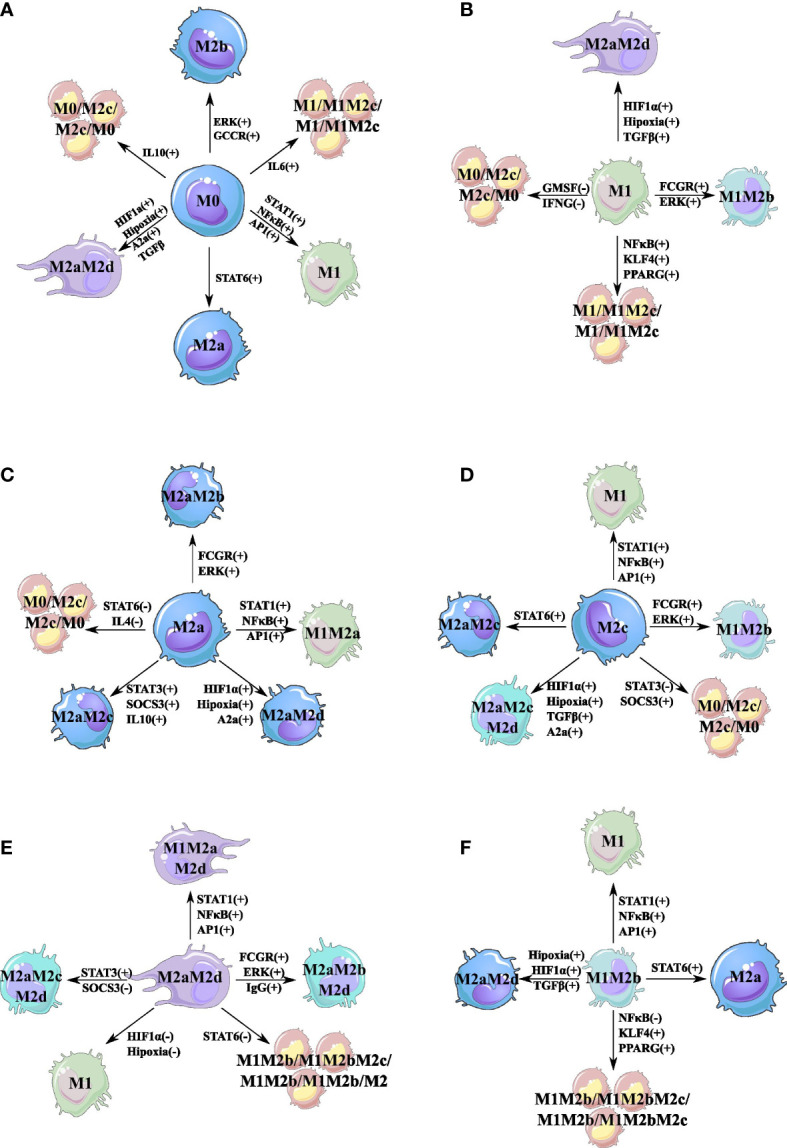
Cell fate map of the macrophage polarization. For each attractor obtained, we changed the node’s value and maintained this perturbation until an attractor was reached. If the attractor transit to another phenotype, we represent this transition with a line linking the original phenotype with the new phenotype obtained by the perturbation. A single perturbation plus sign (+) means that the node was turned off (0), and we turn it on (1). Meanwhile, a minus sign (−) means that the perturbation was on (1), and we turned it off (0). **(A)** Cell fate map for monocyte (M0). **(B)** Cell fate map for M1 macrophage. **(C)** Cell fate map for M2a macrophage. **(D)** Cell fate map for M2c macrophage. **(E)** Cell fate map for M2aM2d macrophage. **(F)** Cell fate map for M1M2b macrophage. Colors represent different states (attractors) of macrophage polarization.

With the purpose of sketching the phenotype landscape, we evaluated the feasible transitions between attractors when a bit-flip perturbation on the state of a node occurs. This perturbation was computationally accomplished by permanently changing the state of a node from 0 to 1, or *vice versa*, of every single attractor. This type of perturbation will determine the one-state neighbors. As **Figure 2** depicts, we have characterized the space of feasible transitions for six single attractors obtained for the TRN of macrophages. We will start by describing the dynamics of the monocyte in response to an inner perturbation (see **Figure 2A**). Our first observation was that the activation of STAT1 or NF-*κ*B in monocytes favors the transition to the M1 phenotype (**Figure 2A**). Furthermore, when we introduce a monocyte into an IL-10 microenvironment (simulated by the activation of IL-10), it transits to a cycle attractor with regulatory capacity. Monocytes can secrete IL-10 and TGF-*β*, both of which independently activate STAT3 (45, 46). In addition, autocrine secretion of the effector of immune secretion TGF-*β* is an essential factor for the maintenance and survival of monocytes; TGF-*β* is also secreted by M2c macrophages (27, 47).

On the other hand, the TFs associated with the pro-inflammatory of the M1 phenotype are NF-*κ*B or STAT1. If we inactivate NF-*κ*B, the M1 macrophage will transit to a cycle attractor M1/M1M2c/M1/M1M2c, where AP-1 is associated with M1 and STAT3 is associated with M2c (**Figure 2B**). AP-1 activation in M1 macrophages is implicated with the production of nitrogen intermediates, IL-6, and TNF-*α* (48), thus developing tumoricidal capacities (49, 50). Instead, the M2c counterpart is associated with the secretion of IL-10, TGF-*β*, and IL-6 (27). The only cytokine in common in the cycle attractor M1/M1M2c/M1/M1M2c is IL-6, which will dictate the oscillations between states, a pleiotropic cytokine with a mechanism of regulating the secretion of cytokines by macrophages. Interferon-γ (IFN-γ) is an important factor because of its pro-apoptotic and anti-proliferative behavior in TME. If we inactivate IFN-γ, the M1 macrophage will transit to a cycle attractor M0/M2c/M2c/M0 losing the anti-tumor properties because of the inactivation of STAT1 (**Figure 2B**) (51). Granulocyte-macrophage colony-stimulating factor (GM-CSF) is implicated with pro-inflammatory components, and if inactivated, it will transit to the M0/M2c/M2c/M0 attractor (**Figure 2B**) (52). Moreover, losing this TF is associated with a naive monocyte, because non-native monocytes are differentiated in the presence of GM-CSF, and naive macrophages are more susceptible to the environmental stimulus (53).

In terms of the M2a macrophage, we noted that as STAT6 is deleted, the macrophage shifts to the cycle attractor M0/M2c/M2c/M0 (**Figure 2C**). Instead, the unfavorable regulatory and pro-tumoral macrophage M2c is activated by STAT3 and the inhibition of SOCS3 (**Figure 2D**). Once we perturb STAT3 in M2C, the macrophage polarizes to a cycle attractor, M0/M2c/M0/M2c. If we activate SOCS3 through STAT1 or NF-*κ*B, it will change the phenotype and enter the same cycle attractor (**Figure 2C**).

From a biological sense, M2aM2d is an unfavorable macrophage because it is associated with tumor-promoting properties by stimulating the production of cytokines and growth factors with pro-proliferative capacity. Our computational analysis postulates that the key TFs that shape M2aM2d are STAT6 and HIF1*α* for the M2a and M2d macrophages, respectively (**Figure 2E**). The M2aM2d macrophage hybrid phenotype is implicated in a regulatory behavior, secreting cytokines and growth factors to enhance tumor growth; this is one of the phenotypes we want to avoid in a hyper-regulatory environment (8). When STAT6 is perturbed, the state of the network transits to the cycle attractor M1M2b/M1M2bM2c/M1M2b/M1M2bM2c. STAT6 can repress the expression of the TF associated with the M1 phenotype (STAT1 and NF-*κ*B) and the M2b (AP-1) macrophage phenotype (27). The M2b macrophage can secrete pro-inflammatory cytokines (IL-6 and TNF-*α*)nd anti-inflammatory cytokines (IL-10) (27, 54). Once we inactivate the expression of STAT6, it allows the expression of the M1M2b (STAT1 or NF-*κ*B mixed with AP-1) hybrid phenotype. Meanwhile, this perturbation tilts the balance to a more pro-inflammatory state and the macrophage generates a cycle attractor that goes back and forth with the regulatory component of the M2c macrophage phenotype, which counteracts the proinflammatory state by secreting IL-10. Finally, another important attractor is the hybrid M1M2b (**Figure 2F**), which has a mixture of favorable and unfavorable properties in a TME. For this hybrid, the important TFs are NF*κ*B and AP-1 activated by IL-1 **β*.* If we inactivate IL-1*β*, it will polarize to a favorable macrophage M1 because AP-1 is no longer activated, and we lose the M2b counterpart. However, if we inactivate NF-*κ*B by inhibiting TNF-*α* in the microenvironment, the hybrid will shift to a cycle attractor, M1M2b/M1M2bM2c/M1M2b/M1M2bM2c, which acts as a reservoir so as not to lose the whole M1M2b hybrid phenotype. If we activate peroxisome proliferator-activated receptor-*γ* (PAR-*γ*) or Kruppel-like factor (KLF4), M1M2b will transit to the same cycle attractors because PPAR-γ or KLF4 will eventually activate STAT6, which, in turn, can inactivate NF-*κ*B developing in the first perturbation previously described. KLF4 and PPAR-*γ* are normally activated in low-oxygen microenvironments as a metabolic response in a hypoxic scenario. Also, these factors are implicated in releasing growth factors and cytokine associated with an M2a macrophage trying to feed cancer cells in an adverse scenario that allows them to thrive.

## Discussion and future directions

In recent years, cancer has been studied as a complex system by taking into account the interacting TFs, cytokines emitted at different states, and other intracellular signals coming from the TME (55–57). Nevertheless, to our knowledge, there are very few studies that explore the role that immune cells have in cancer progression, specifically the macrophage. Macrophages are the most abundant cells in tumors and are associated with poor clinical outcomes (58); therefore, understanding their mechanisms of adaptation to constant signaling in a TME can have important therapeutic implications. Moreover, some studies have used this cell as a driver of tumor cell growth promoter (59) and favoring the metastatic process (60). All these studies on cancer make it evident that the microenvironment is a factor that can promote or repress cancer growth depending on the specific signaling profile. Based on this idea, the control of the activity of the macrophage has been considered an appealing strategy to diminish its lethal effects (37, 61, 62).

Boolean modeling is an excellent approach to understanding the mechanisms that drive macrophage polarization in a TME. Despite its mathematical simplicity, Boolean modeling of macrophage polarization may highlight certain patterns of expression that may help us understand the macrophage phenotypes whose function in a TME remains unknown. Notably, the single and hybrid attractors obtained from the macrophage TRN have been useful to associate the different phenotypes with those experimentally validated, especially M1 and M2 states. However, cycle attractors in the Boolean modeling approach have been mostly disregarded in a variety of studies because they have been considered mathematical artifacts without any biological relevance. Recently, one article has focused on these types of attractors and pointed out the biological implications of cycle attractors in a model of macrophage polarization (17). Here, we postulate another idea that supports their biological relevance. By accomplishing a genetic perturbation using a previous TRN in macrophages (8), we observe that the cyclic attractors can be seen as a reservoir of states for balancing the pro- and anti-inflammatory response in a TME. Our study contributes additional evidence that oscillatory phenotypes could have a relevant role in maintaining the pro- and anti-inflammatory balance in any type of immune response, including cancer (63). For example, M1 and M1M2b are macrophages with favorable tumor-eliminating outcomes; however, to avoid a hyper-inflammatory state, a perturbation in the TME will help to reduce entering this state associated with complications in patients (55). The same pattern occurs for the macrophage phenotypes with unfavorable tumor-promoting outcomes; the TME induces a cyclic attractor in the macrophage to delay the hyper-regulation in the environment. A hyper-regulation state is associated with the proliferation of tumors and immune evasion due to the action of secreted cytokines (25, 56), which is why sustained regulation of macrophages should be inhibited by oscillations.

From our analysis, we hypothesize that the cycle attractors could have therapeutic implications by circumventing hyper-inflammatory and regulatory states. In particular, three cycle attractors were obtained in our analysis; one of them involves the monocyte (M0) and the M2c state: M0/M2c/M0/M2c. The second attractor is defined by the states M1 and the hybrid state M1M2c: M1/M1M2c/M1/M1M2c. Finally, the last attractor comprises the hybrid states M1M2b and M1M2bM2c: M1M2b/M1M2bM2c/M1M2b/M1M2bM2c. One important aspect to take into account is the nature of the phenotype of where it is before reaching the cyclic attractor in response to the disturbance. The most abundant cycle attractors that are present in four of the six major attractors are depicted in **Figure 2**. This attractor transits from the monocyte (M0), the pro-inflammatory with cytotoxic activity M1 macrophage, and the regulatory and tissue recovery macrophages M2a and M2c. The M0/M2c/M0/M2c cyclic attractor emerges by modifying the IL-10 from a monocyte and is associated with the secretion of IL-10 and TGF-*β* cytokines and with the survival and continuity of monocytes in the TME. In addition, this cycle attractor may be associated with higher infiltration of macrophages in the microenvironment, which is implicated in a lower survival rate (27, 47, 64). Monocytes that are continuously recruited because of the cycle attractor are implicated in resistance to treatment and enhance immunosuppression (65–67). Moreover, this cycle attractor is implicated with a higher abundance of monocytes in the blood, a bad prognosis for cancer patients (68, 69). The same attractor emerges from the pro-inflammatory and cytotoxic M1 phenotype. The difference between both sources radicates on the cytokines or the growth factor that triggers the final state. Regardless of the necessity of the M1 phenotype, it has been demonstrated that an uncontrolled pro-inflammatory state may be associated with the enhancement of cell death due to the induction of the cytokine storm (70). Accordingly, with our *in silico* study, the M0/M2c/M0/M2c attractor appears when GM-CSF and IFN-γ are absent in the microenvironment. GM-CSF is a double-edged sword towards tumor progression. It is implicated in tumor cell elimination; however, in a high dose, it is associated with tumor resistance by modifying the TME (71). A continuous expression of this stimulator has been associated with a hyper-inflammatory scenario due to the imbalance in the secretion of inflammatory cytokines and chemokines (72, 73). Through inhibition of GM-CSF expression, we can avoid a hyper-inflammatory state leading the system to enter into a cycle attractor with anti-inflammatory cytokines to try to maintain homeostasis and circumvent therapeutic resistance (74, 75). Given that IFN-γ has an anti-proliferative cytotoxic response against tumor cells, it is an excellent candidate for immunotherapy (51, 76). Nevertheless, the TFs in macrophages that secrete IFN need to be continuously activated; otherwise, NF-*κ*B or AP-1 may maintain the secretion of pro-inflammatory cytokines and propitiate the cytokine storm. To avoid the cytokine storm induced by immunotherapy (74, 75), the network enters a cycle attractor. We suggest that this dynamic state is a response to the loss of IFN- d the secretion of anti-inflammatory cytokines. In summary, by entering this cycle attractor, the macrophage tries to prevent a hyper-inflammatory state by cycling between anti-inflammatory and pro-inflammatory (AP-1 activated) phenotypes. A microenvironment where the balance is tilted to an anti-inflammatory state is a scenery that is not desirable in cancer. It has been demonstrated that this state is detrimental to the patients because it enhances the survival and growth rate of tumor cells (8, 68). Forthwith, we will focus on the importance of the cycle attractor M0/M2c/M0/M2c when the nature of the attractor has anti-inflammatory properties. The M0/M2c/M2c/M0 that emerges from the M2a derives from a macrophage associated with the secretion of growth factors implicated in recuperating tissue by remodeling with fibrogenic inflammatory cells around a tumor. The latter transition is due to the fact that IL-4 is absent in the microenvironment and consequently inactivates STAT6 (which is inhibited in the macrophage). Thus, the Th2 response does not exist in the environment. Hence, the emergence of this cycle attractor is of great importance because it prevents the hyper-regulatory state of the macrophage (77). Because IL-4 activates the macrophage, it generates positive feedback with a Th2 response allowing the secretion of cytokines implicated in inhibiting the cytotoxic function and presence in the microenvironment of IFN-*γ* (78–80) Finally, the last transition for this cycle attractor is from an M2c hyper-regulatory macrophage; when it loses the expression of STAT3, it transits to a cycle attractor with the survival of monocytes. We can observe that these macrophage phenotypes enter a cycle attractor that will not avoid a hyper-regulatory state, instead with continuous recruitment of monocytes and their survival. This cycle attractor may be implicated with the capacity of macrophages to induce immune suppression by the secretion of cytokines and chemokines that enhance a Th2 response (81, 82) and enhancement of metastasis (83).

M1/M1M2c/M1/M1M2c derives from two important macrophages: the monocyte and the M1 macrophage. The cycle attractor emerges when IL-6 is present in the microenvironment, a pleiotropic cytokine with the capacity to activate not only TF associated with a pro-inflammatory phenotype but also TF associated with an anti-inflammatory behavior. The activation of this cytokine generates the cycle attractor M1/M1M2c/M1M2c, where AP-1 and STAT3 are the TFs activated in this cycle. The cycle goes back in forth with the secretion of proinflammatory cytokines (like IL-6, TNF-, IL1-*β*, and the tumor-eliminating cytokine IL-12) balanced with the secretion of IL-10 to try to diminish the inflammatory counterpart, preventing a hyper-inflammatory state. When M1/M1M2c/M1M2c shifts from the M1 macrophage, it has the same behavior to prevent a hyper-inflammatory state; the only difference is the TF involved. KLF4 and PPAR-*γ* are TFs associated with the secretion of anti-inflammatory cytokines (84–86); PPAR-*γ* develops negative feedback with AP-1 inhibiting the secretion of pro-inflammatory cytokines (49), allowing instead the secretion of IL-10 and Arg1, which is associated with an immunosuppressive function (87). Meanwhile, KLF4 is implicated with an anti-inflammatory but STAT6-dependent response, favoring an IL-4-type macrophage (M2a) and a Th2 response in a TME (88, 89).

M1M2b/M1M2bM2c/M1M2b/M1M2bM2c only comes out from an unfavorable hybrid macrophage M2aM2d due to its pro-tumor behavior and the ability to function in a hypoxic condition (90) and the M1M2b with the capacity to eliminate tumors. The cycle attractors that emerge from M2a are correlated with a hyper-regulatory state, leading to angiogenesis and tumor fibrosis. The TF associated with this behavior is STAT6; when inhibited, it polarizes a cycle attractor that limits pro-inflammatory secretion at certain periods in the cycle. The periods where pro-inflammatory cytokines are secreted will avoid the hyper-regulatory state, and it may tilt the balance to a more tumor-eliminating phase but without surpassing the hyper-inflammatory state (this may be achieved with the presence of M2c in the cycle). Meanwhile, the cycle attractor that emerges from M1M2b can be regulated by inhibiting NF-*κ*B and cycle back and forth with pro-inflammatory and anti-inflammatory cytokines. This transition between the hybrid and the cycle attractor is of great importance to prevent a hyper-inflammatory state associated with tissue damage and tumor growth.

We concluded that cycle attractors act as reservoirs for refueling a balance in the population when the TME reaches a hyper-inflammation or a regulatory state. They can also behave as bystanders in the TME, waiting for the perturbation to cease and prevent hyper-inflammation or hyper-regulation. Interestingly, we can take advantage of this cycle attractor to incline the balance to a more tumor-eliminating phenotype by mechanisms of immunotherapy. Altogether, we conclude that cycle attractors of macrophage polarization are biologically relevant because they describe the adaptability of macrophages in transducing the TME signals and function based on that response. Based on this perspective article, we recommend to not ignore these attractors and to carefully evaluate and start designing experiments to classify their possible biological relevance. Finally, we highlight some drawbacks that should be addressed in future works to improve the results discussed here. In terms of limitations, although we have simplified the spatial distribution of macrophages and their effects on transcriptional regulation, this assumption is inappropriate in real systems. For instance, a specific population of macrophages is located adjacent to endothelial cells, which help the secretion of factors implicated with the regulation of angiogenesis (91, 92). Thus, Boolean modeling with the spatial components and the heterogeneous composition of cells is a challenge that needs to be addressed in future studies. To contribute to these last aims, single-cell and spatial RNA seq technologies are fundamental technologies that can help understand the heterogeneity and try to generate a nomenclature of macrophage diversity in a TME (93–95). Furthermore, by applying these technologies in macrophage polarization, we expect to have valuable information about the repertoire of phenotypes to confirm our main hypothesis: cycle attractors are a means to respond to a specific perturbation or to avoid a hyper-inflammatory or regulated state.

Lastly, we highlight two strategies to induce a transition from a phenotype within a cycle state to a state that favors tumor elimination: reprogramming the environment or engineering the transcriptional regulation of the macrophages. The first tactic is achieved by modifying the environment and creating the secretion of the opposing cytokines to favor tumor elimination for some time without entering the hyper-inflammation or regulation phase. The second strategy is focused on transcriptional activity, for example, the inactivation of STAT6 or STAT3 to recover phenotypes in a tumor-eliminating state. The validation of the conjectures obtained from the Boolean approach is subject to experimental assessment in the near future.

## Data availability statement

The original contributions presented in the study are included in the supplementary material. All code used in this paper are freely available at https://github.com/resendislab/Cyclic_Attractors.

## Author contributions

UA-P and OR-A conceived and designed the mathematical model. UA-P and AV-J performed all computational analyses and analyzed the data. MM-G and AV-J helped with the analyses of the data. All authors contributed to the article and approved the submitted version.

## Funding

ORA thanks the financial support from CONACYT (Grant Ciencia de Frontera 2019, FORDECYT-PRONACES/425859/2020), PAPIIT-UNAM (425859), and an internal grant from the National Institute of Genomic Medicine (INMEGEN, México). UA-P would like to thank the financial support from CONACYT (CVU: 774988).

## Acknowledgments

UA-P is a doctoral student from Programa de Doctorado en Ciencias Biológicas of the Universidad Nacional Autónoma de México (UNAM). This paper is part of the requirements to obtain the degree of doctor in the said program. UA-P also received a fellowship (CVU: 774988) from Consejo Nacional de Ciencia y Tecnología (CONACYT).

## Conflict of interest

The authors declare that the research was conducted in the absence of any commercial or financial relationships that could be construed as a potential conflict of interest.

## Publisher’s note

All claims expressed in this article are solely those of the authors and do not necessarily represent those of their affiliated organizations, or those of the publisher, the editors and the reviewers. Any product that may be evaluated in this article, or claim that may be made by its manufacturer, is not guaranteed or endorsed by the publisher.
